# Full-length transcriptome of in *Medicago sativa L.* roots in response to drought stress

**DOI:** 10.3389/fgene.2022.1086356

**Published:** 2023-01-04

**Authors:** Zhihong Fang, Jianning Liu, Xinming Wu, Yan Zhang, Huili Jia, Yonghong Shi

**Affiliations:** College of Grassland Science, Shanxi Agricultural University, Jinzhong, Shanxi, China

**Keywords:** *Medicago sativa L.*, root, full-length transcripts, drought stress, gene expression

## Abstract

**Background:** Alfalfa (*Medicago sativa L.*), serves as a legume with high drought tolerance, is a major forage crop with a high biomass of production. However, the molecular mechanism of Alfalfa in response to drought stress are still unclear.

**Results:** We constructed the first full-length transcriptome for Alfalfa root. 21.53Gb clean data were obtained by further data filtering, in which incorporate 566,076 reads of Insert (ROI), and 409,291 full length reads non-Chimeric (FLNC) sequences. Combined with second-generation sequencing (SGS), there were 2615, 6011, and 4617 differentially expressed genes (DEGs) in three comparisons. KEGG pathway analysis showed enrichment of ribosome, glutathione metabolism, and biosynthesis of amino acids are among the DEGs. The majority of transcription factors (TFs) from DEGs were AP2/ERF-ERF (37), C2H2 (32), and bHLH (22) bZIP (22), followed by C3H (19), MYB (18), WRKY (18), GRAS (16), and NAC (15). 32 C2H2 genes were differentially expressed in three groups. In addition, TFs annotated as C3H (19), MYB (18), GRAS (16), and NAC (15) also changed significantly in expression in the three comparisons. We found 24 genes participate in the abscisic acid (ABA) and auxin hormone signaling pathway in response to drought stress, and monitored the expression patterns of these related genes.

**Conclusion:** The present study enhanced our understanding of the genetic diversity and complexity, and provides greater insight into the fundamental transcriptome reprogramming of Alfalfa under drought.

## Introduction

Alfalfa (*Medicago sativa L.*) serves as a crucial leguminous forage crop around the world, which is a sort of highly productive perennial forage species (Fu et al., 2015). Alfalfa is also the artificial forage with the largest planting area and the widest distribution in China (Wang et al., 2016). Alfalfa also has strong adaptability, rich nutrition and excellent quality (Panchenko et al., 2017). Its stems and leaves are rich in protein, minerals, multi-vitamins and carotene, and it is an important source of protein feed and forage for various livestock (Jiang et al., 2016). Moreover, it plays an important role in improving soil, fertilizer and soil and water conservation (Zhang et al., 2007). Alfalfa is also known as the “king of herbage” (Ma et al., 2016). Its life span can be up to 30 years, and its field cultivation and utilization life can be up to 7–10 years, with strong reproducibility (Shu et al., 2016). Abiotic stress (high salt, low temperature, drought, etc.) is a common environmental factor in nature, which severely restricts the growth and development of plants (Sah et al., 2016). At present, the effects of abiotic stress, especially drought stress, on crops have become the bottleneck of agricultural development in many areas (Choudhury et al., 2017). Therefore, it is of great significance for improving the tolerance of crops on drought stress to study the stress resistance of Alfalfa At present, the genome and transcriptome information of Alfalfa roots under many abiotic stresses has not been explored, which greatly hampers the study of the underlying molecular mechanisms of drought stresses on Alfalfa growth and development.

The transcriptome is the complete set of transcripts for certain type of cells or tissues in a specific developmental stage or physiological condition (Foley et al., 2015). Transcriptome analysis can reveal the gene expression levels of organisms, structural variation, discovery of new genes (Grabherr et al., 2011). The research methods and platforms of transcriptome are undergoing rapid changes, and bioinformatics analysis is also gradually improved. The single-molecule real-time (SMRT) sequencing technology from Pacific Biosciences (PacBio) could more quickly and accurately provide more transcriptional information of organisms (Yanhu et al., 2015; Nakano et al., 2017). Therefore, this technology has become a better alternative for full-length cDNA molecular sequencing, which has been widely applied in whole-transcriptome profiling of human and other species. Within the past few years, the rapid development of second-generation sequencing (SGS) has also led to the increase of data throughput and read length, and simultaneously brought down substantially the sequencing cost (Zhang et al., 2016). This made new breakthrough in the area of biology and ushered the medical genetics into a new era.

In present study, we utilized the SMRT sequencing to generate and identify full-length transcriptome of Alfalfa roots under drought stress. According to the transcriptome data, we analyzed the full-length transcriptome sequences, transcription factor, complete coding sequence (CDS), and transcript functional annotation. In addition, we combined PacBio sequencing and SGS technology to further identify the differentially expressed genes and analyze the functional annotation in Alfalfa roots under drought stress. Our study may be a valuable resource for further investigation of Alfalfa roots under water deficit.

## Materials and methods

### Plant cultivation and drought stress treatment

Alfalfa plant was provided by Institute of Animal Husbandry and Veterinary Science, Beijing Academy of Agricultural Sciences, Beijing, China. Plants grew in the soil in the greenhouse under a 16 h light/8 h dark cycle, 25°C ± 1°C and 80 ± 5% relative humidity, and watering twice a week. After 28 days, the ensuing plant were then exposed to drought stress for 0, 48 and 72 h, and roots were collected from plant. There were three biological replicates for each time point. A biological replicate contain mixed sample of three plant roots, and stored at −80°C for further analysis.

### RNA sample preparation

Total RNA was isolated by using TRIzol (Invitrogen, Carlsbad, CA, United States) following the manufacturer’s instructions. 1% agarose gels were used to detect RNA degradation and contamination. NanoDrop ND-1000 spectrophotometer (NanoDrop Technologies, Rockland, DE, United States) was used to analyze the purity concentration of RNA (OD260/280). The RNA integrity was evaluated by the RNA Nano 6000 Assay Kit on the Agilent Bioanalyzer 2100 system (Agilent Technologies, CA, United States). All total RNA samples were used for the following experiments.

### Library preparation and SMRT sequencing

The poly (T) oligo-attached magnetic beads were utilized to purify the mRNA from the mixed RNA. The divalent cations were applied to manage the fragmentation under elevated temperatures. The full-length cDNA of mRNA was synthesized by using SMARTer™ PCR cDNA Synthesis Kit (Clontech, CA, United States). The remaining overhangs were used generate blunt ends by exonuclease/polymerase activities. And then BluePippin (Sage Science, Beverly, MA, United States) was used to screen full-length cDNA fragments and construct cDNA libraries. The filtered cDNAs were re-amplified by PCR assay, and the distribution of fragment size was assessed by the Qubit fluorometer (Life Technologies, Carlsbad, CA, United States). Full-length cDNA ends were repaired, SMRT joints were connected. The cDNAs were re-screened to obtain the sequencing library by BluePippin. The libraries were evaluated quantitatively by a Qubit2.0 DNA kit (Life Technologies, China), Size of the libraries was detected by Agilent 2100. Finally, cull-length transcriptome sequencing was performed using PacBio Sequel. All raw sequencing data for the alfalfa *sativa* Raw sequence reads have been deposited at the Bio Project under accession code PRJNA531296. And the SRA records are accessible with the link (https://www.ncbi.nlm.nih.gov/sra/PRJNA531296).

### Isoform sequence clustering

Iterative sequence clustering was analyzed by SMRT Analysis software with Iterative Clustering for Error Correction (ICE) algorithm. Similar sequences were clustered into clusters, each of which yields a consensus isoform. The consistent sequences in each cluster were corrected by quiver application. Finally, we obtained high-quality transcripts (HQ, high-quality isoforms) with accuracy greater than 99%.

### Functional annotation

BLAST software (version 2.2.26) (Altschul et al., 1997) was used to compare the non-redundant transcripts with databases of NR, Swissprot, Gene Ontology (GO) (The Gene Ontology Consortium, 2019: 20 years and still GOing strong 2019), Pfam (Mistry et al., 2021), and Kyoto Encyclopedia of Genes and Genomes (KEGG) (Du et al., 2014) to obtain the annotation information of transcripts.

### Next-generation sequencing and quantification of gene expression levels

Total RNA was digested by DNase I (NEB, Frankfurt, Germany). The sample was purified by using Agencourt RNA Clean XP Beads, and then fragmented. First Strand Master Mix and Super Script II reverse transcription (Invitrogen) was used to synthesize First-strand cDNA, and Second Strand Master Mix was used to generate the second-strand cDNA. After end repairing, the cDNA fragments were amplified by using PCR Master Mix. Finally, the library was quantitatively quantified by the Agilent 2100 bioanalyzer instrument and RT-qPCR. And the Illumina HiSeq. 2000 System was used to qualify the libraries.

The expression levels of the unigenes in the nine samples were calculated by RNA-Seq quantification analysis. Raw data (raw reads) of fastq format were first processed through in-house Perl scripts. At the same time, the Q30, GC content and sequence duplication level of the clean data were calculated. All downstream analyses were based on clean data with high quality. The transcriptomes of the samples were used as a reference to screen the clean reads. Only reads with a perfect match or one mismatch were further analyzed and annotated based on the reference transcriptome. Tophat2 tools were used to map the full-length transcriptome data.

Quantification of gene expression levels was estimated by the fragments per kilobase of transcript per million fragments mapped. For the samples with biological replicates, prior to differential gene expression analysis, for each sequenced library, the read counts were adjusted by the edgeR program package through one scaling normalized factor. Differential expression analysis of samples was performed using the EBSeq R package. The resulting false discovery rate (FDR) was adjusted using the PPDE (posterior probability of being DE). The FDR<0.05 and |log2 (fold-change) | ≥1 were set as the threshold for significant differential expression.

### Validation of gene expression by qRT-PCR

Seven genes were screened for validation using quantitative real-time PCR (qRT-PCR). The LightCycler®480 II Real-Time System (LightCycler®480 II cycler, Roche, Carlsbad, CA, United States) was utilized to perform the qRT-PCR. The thermal profile for the PCR amplification was 95°C for 5 min, followed by 40 cycles of 10 s at 95°C and another 40 cycles at 60°C for 30 s. According to the instruction’s protocol, all the PCR reactions were performed using the HieffTM qPCR SYBR Green Master Mix (No Rox) (Yeasen Biotech Co., Ltd., Shanghai, China). All the qRT-RCR analyses were conducted with three technical, and three biological replicates were conducted in all qRT-RCR analyses. According to the 2-△△CT method, we calculated expression level of different genes to the control (Livak and Schmittgen 2001).

### Statistical analysis

Experimental data were all presented as mean ± SEM, and analyzed with Graghpad (Ver. Prism 7, GraphPad Prism Software, La Jolla, CA, United States). The results were analyzed using Student’s t test and one-way analysis of variance analysis as appropriately, and *p* < 0.05 was considered as statistically significant.

## Results

### Full-length RNA-sequencing and functional annotation

To explore the molecular mechanisms underlying drought response, the roots of the differentially-treated plants was employed to perform sequencing. The FLNC length distributions of each size bins and 1–6 k size bin were in accordance with anticipation (Supplementary Figures S1A, B). According to the ROI classification for 1-6k size, the percentage of the filtered short reads was 6.2%. The percentage of the full-length (chimeric) was 1%. The percentage of the full-length (non-chimeric) was 73.2%. The percentage of the non-full-length (no poly-A) was 11.6% and the percentage of the non-full-length (no primer) was 8.9% (Supplementary Figure S1C). 21.53Gb clean data were obtained by further data filtering, in which incorporate 566,076 ROI, and 409,291 FLNC sequences.

The IsoSeq module of SMRT Analysis software was used for clustering analysis of full-length sequences, and 194,286 consistent transcripts were obtained. A total of 41,248 high-quality transcripts were corrected by non-full-length sequences, and third-generation low-quality data were corrected by second-generation data. A total of 81,017 transcripts were obtained by de-redundancy analysis of high-quality transcripts and corrected transcripts.

Finally, 77,221 of the 81,017 non-redundant transcripts could be annotated to different databases. Specifically, 32,297, 34,349, 48647, 62168, 54521, and 55044 isoforms were respectively annotated to the COG, KEGG, KOG, Pfam, Swiss-Prot, and GO databases (Supplementary Table S1).

### Comparative analysis of DEGs

**FIGURE 1:**
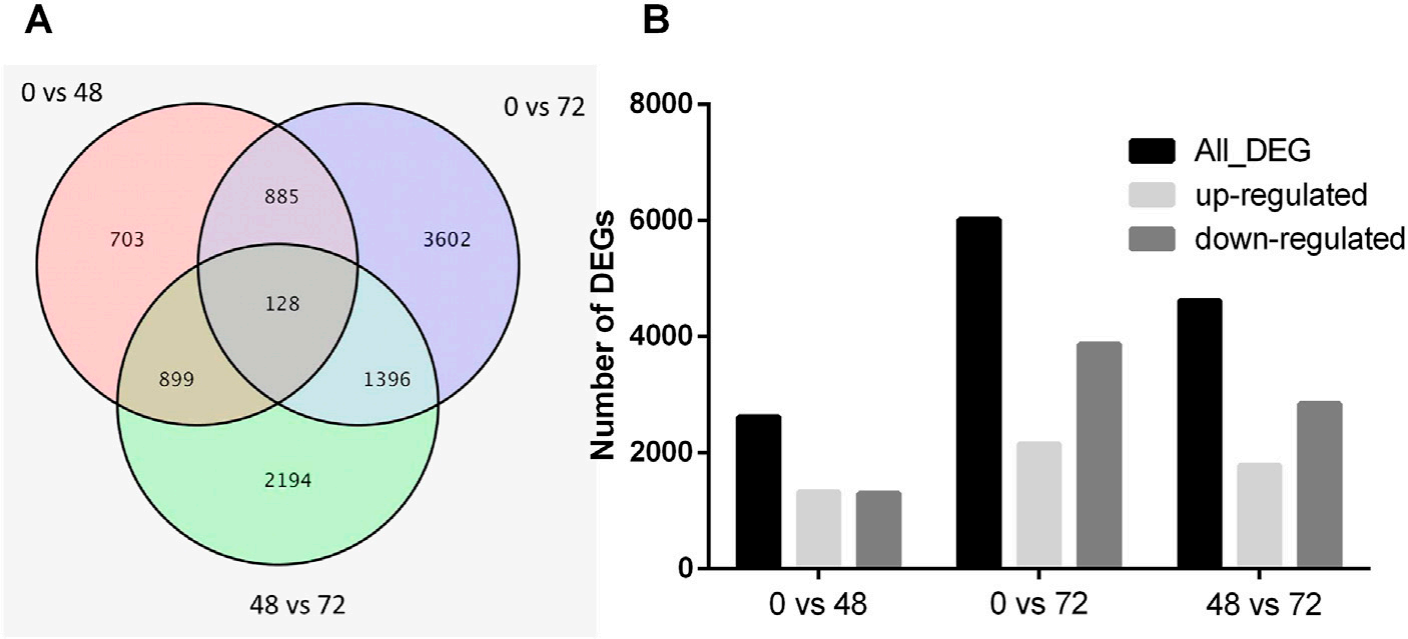
The number of DEGs in comparison group. **(A)** Veen figure exhibits unique and common DEGs in comparison group. **(B)** The histogram illustrating significantly upregulated, downregulated and all DEGs.

To further elucidate the drought-induced transcriptomic variations in roots, 9,807 DEGs (Figure 1A) were screened based on the following criteria: FDR (≤0.01) and log2 values (≥2 or ≤0.05). The results indicated that the drought responsive transcriptomic program is predominant in the roots.

There were 2615, 6011, and 4617 DEGs in these three comparisons (Figure 1B). In addition, 1320 genes were up-regulated and 1295 genes down-regulated in the 0 h vs. 48 h, and 2146 genes were up-regulated and 3865 genes down-regulated in the 0 h vs. 72 h, while 1779 genes were up-regulated and 2838 genes down-regulated in the 48 h vs. 72 h. Furthermore, 128 genes were differentially expressed among the 0 h vs. 48 h, 0 h vs. 72 h, and 48 h vs. 72 h (Figure 1A).

**FIGURE 2:**
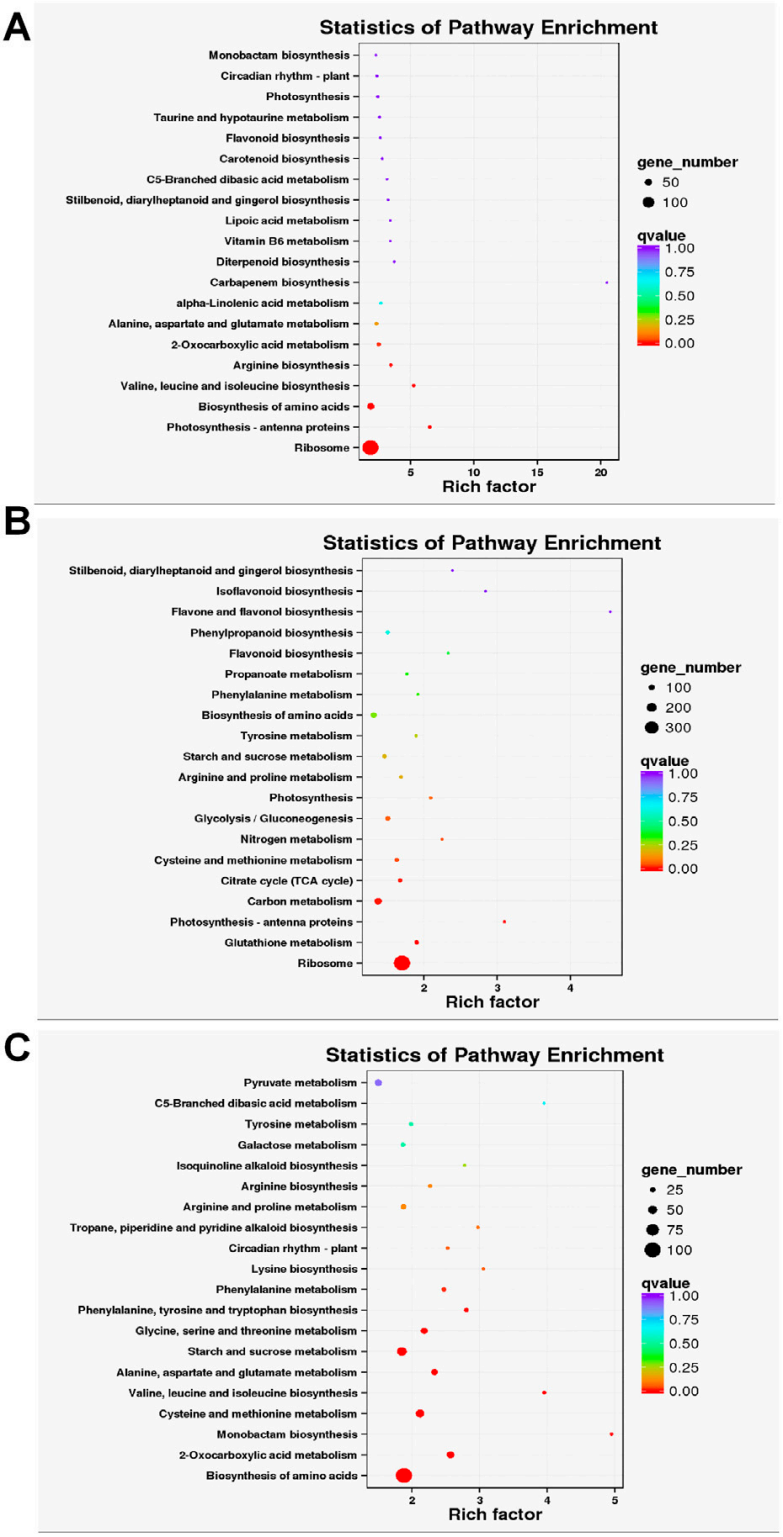
KEGG enrichment analysis of DEGs in 0 vs. 48 h **(A)**, 0 vs. 72 h **(B)** 48 vs. 72 h **(C)**, which showed only the top 20 pathways with the most significant enrichment.

The DEGs from the three comparisons were also analyzed for their KEGG function. At 48 h, the DEGs were significantly enriched into “Ribosome”, “Photosynthesis—antenna” and “Biosynthesis of amino acids” pathways (Figure 2A). Interestingly, at 72 h after drought treatment, the DEGs were significantly enriched into “Ribosome”, “Glutathione metabolism” and “Photosynthesis—antenna” (Figure 2B). Biosynthesis of amino acids, 2-Oxocarboxylic acid, and monobactam biosynthesis were prominently enriched pathway for DEGs of 48 h vs. 72 h (Figure 2C).

### Identification of TF genes involved in drought stress

**FIGURE 3:**
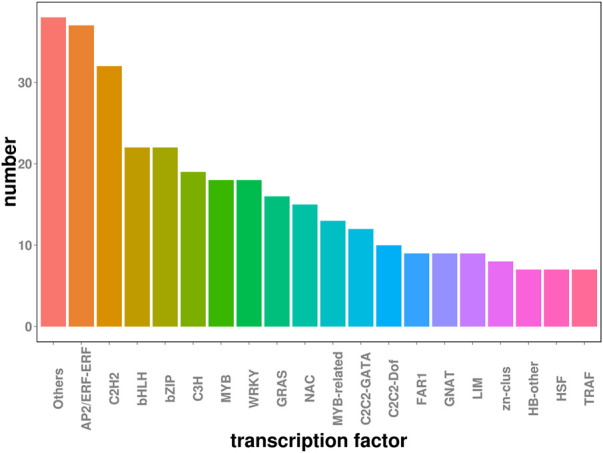
Identification of TFs in DEGs. The horizontal axis represents the names of transcription factors and the vertical axis lists the number of transcription factors.

**FIGURE 4:**
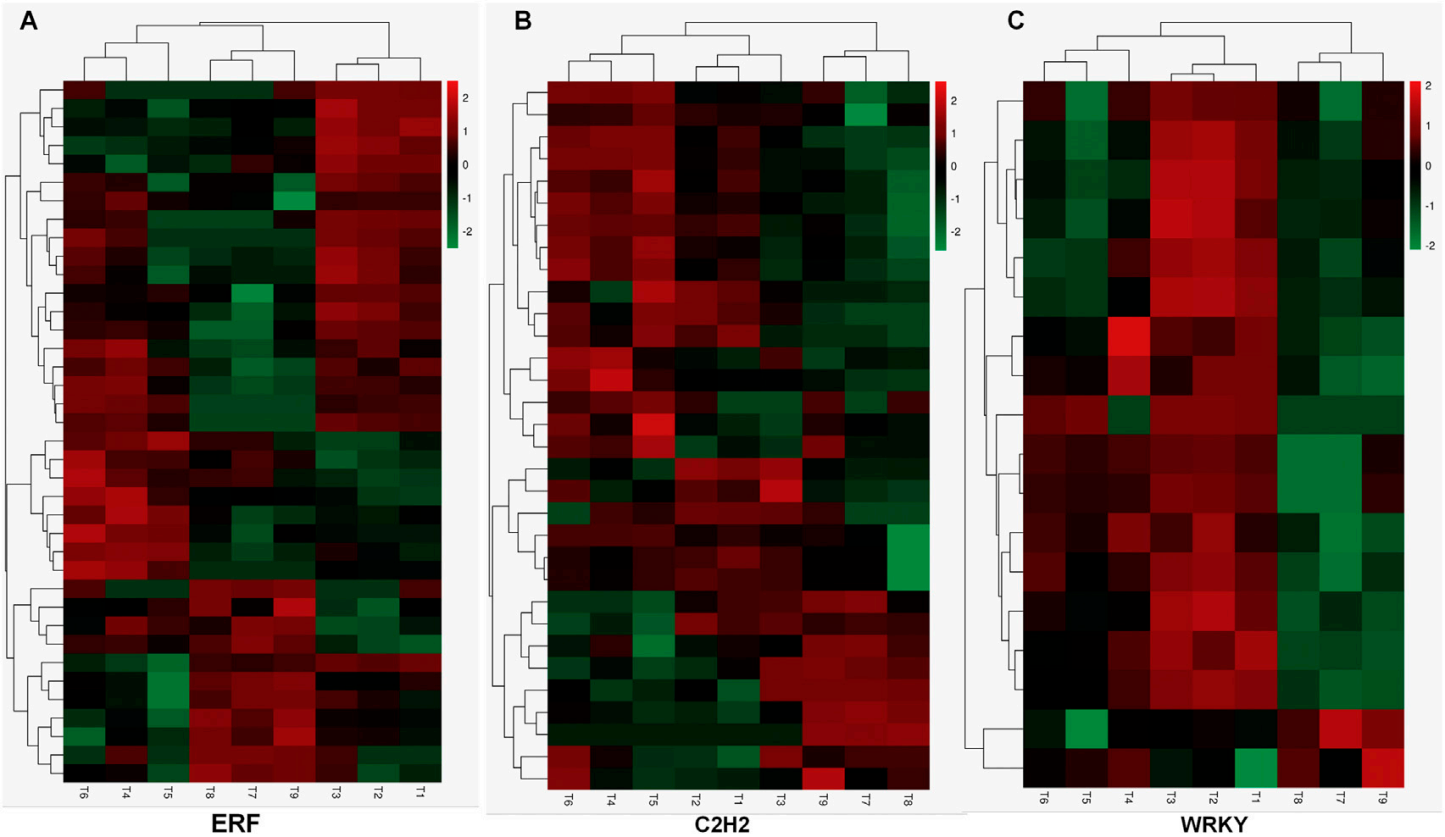
Heat-map showing relative expression levels of ERF **(A)**, C2H2 **(B)**, and WRKY **(C)**. Red represents high expression and green corresponds to low expression.

Transcription factors are a class of proteins that significantly regulate plant growth and development and play an important role in plant defense and stress responses. Figure 3 shows that the majority of TFs from DEGs were AP2/ERF-ERF (37), C2H2 (32), and bHLH (22) bZIP (22), followed by C3H (19), MYB (18), WRKY (18), GRAS (16), and NAC (15). Among these 416 TFs, AP2/ERF-ERF (37) were annotated as potentially participating in plant hormone signal transduction through transcriptome enrichment analysis (Supplementary Table S1). Meanwhile, ERF and WRKY also conduct KEGG enrichment pathway analysis (Supplementary Table S1) ERF (Figure 4A, Supplementary Table S2) and C2H2 genes (Figure 4B, Supplementary Table S3) were identified, and differentially expressed in three groups. Genes annotated as WRKY (18) had significantly decreased levels in 48h and 72 h relative to 0 h (Figure 4C, Supplementary Table S4). In addition, TFs annotated as C3H (19), MYB (18), GRAS (16), and NAC (15) also changed significantly in expression in the three comparisons.

### DEGs involved in ABA and auxin hormone biosynthesis and signaling pathways

24 genes were found to participate in the ABA and auxin hormone signaling pathway in response to drought stress through screening and Studied (Supplementary Table S2). We monitored the expression patterns of these related genes in transcriptome data.

*IAA-leucine resistant 2*, *Auxin-responsive protein SAUR50* and *Auxin-responsive protein SAUR32* were down-regulated in 0 h vs. 48 h. Two bZIP transcription factor (*c367806. graph_c0* and *c361256. graph_c1*) and 1 *Auxin-responsive protein SAUR36* were significantly up-regulated in 0 h vs. 48 h. bZIP transcription factor, *ABA-responsive protein ABR18*, 2 *Auxin-responsive protein SAUR50*, *Auxin-binding protein T85* and *Auxin-induced protein X10A* were up-regulated in 0 h vs. 72 h. 2 auxin associated protein, *Auxin-responsive protein SAUR32* and *IAA-leucine resistant 2* were down-regulated in 0 h vs. 72 h.

### Validation of gene expression by qRT-PCR

**FIGURE 5:**
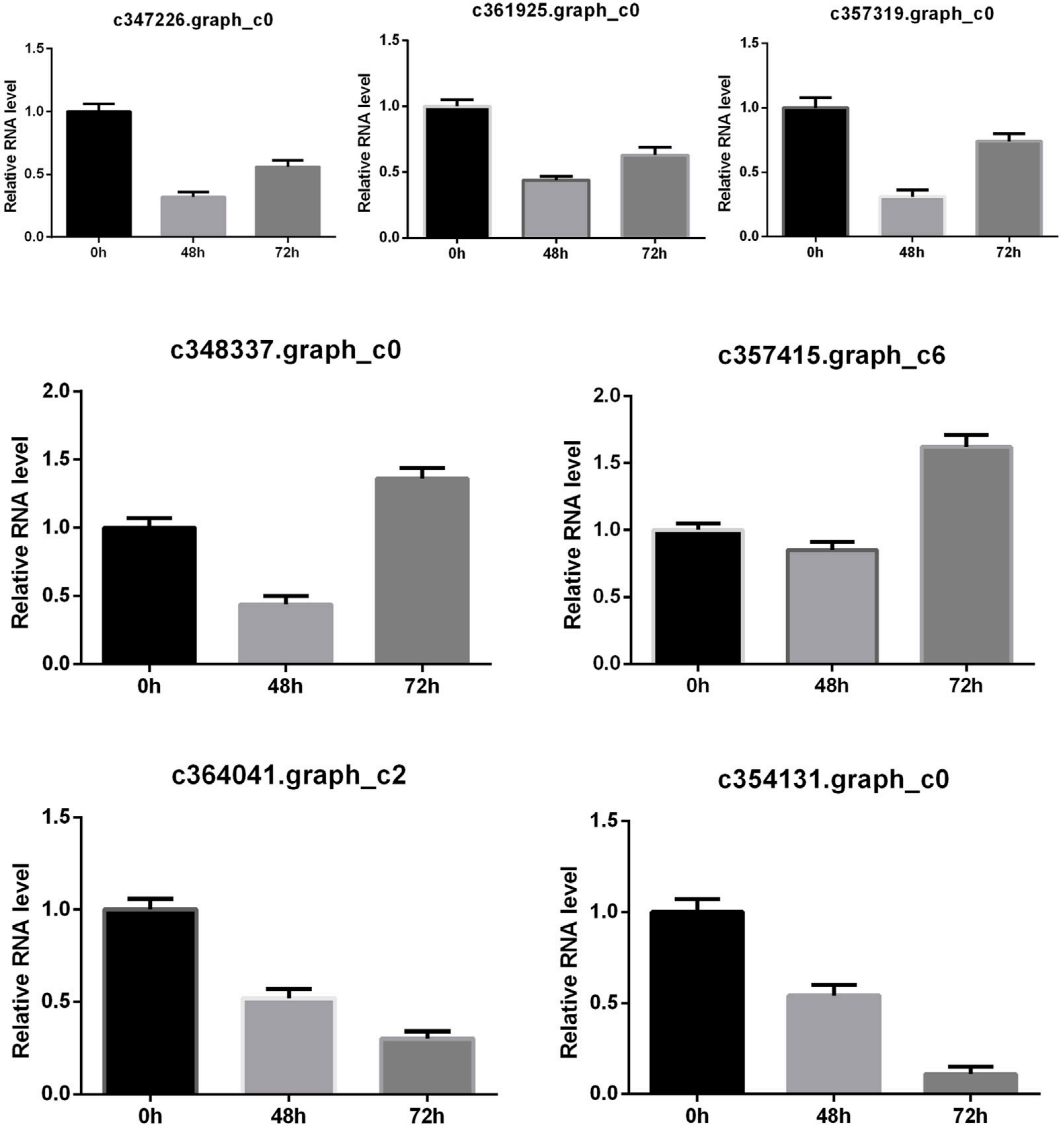
QRT-PCR verification of DEGs. The relative gene expression levels at different periods.

To evaluate the accuracy and reproducibility of RNA-Seq data, seven including 3 EGFR, 2 C2H2, and 2 WRKY genes were selected for qRT-PCR verification. The qRT-PCR confirmed that the expression tendency of these seven genes was highly consistent with the RNA-Seq results (Supplementary Table S5, Figure 5). Thus, the transcriptome data used for DEG analysis were reliable.

## Discussion

Alfalfa, serves as a crucial leguminous forage crop around the world, plays an important role in improving soil, fertilizer and soil and water conservation. However, its growth is often suffered from abiotic stresses like high temperature, salinity stress and drought stress. Therefore, it is crucial to clarify the molecular mechanisms underlying drought stress and adaptability in Alfalfa in order to found candidate genes for reaction mechanism.

We explored the transcriptomes of roots exposed to different drought stress conditions by full-length sequencing. We obtained a total of 81,017 genes including 77,221 annotated genes more than the same reported (He et al., 2021). In other words, the transcriptomic data was evidently abundant compared to the known related data, and can further augment the existing Alfalfa annotation. In response to drought stress, ABA promptly activates stomatal closure path to prevent water deficit, which augment adaptation to prolonged drought. ABA-induced leaf senescence and abscission allows survival during extreme drought conditions (Li et al., 2021). Previous studies found water deficit can quickly triggers the production of ABA, which result in upregulation of numerous ABA-inducible genes (Yamaguchi-Shinozaki and Shinozaki 2006). The ABA-responsive element (ABRE) is the major cis-element for ABA-responsive gene expression. ABRE-binding protein (AREB)/ABRE-binding factor (ABF) transcription factors (TFs) regulate ABRE-dependent gene expression (WRKY transcription factors: key components in abscisic acid signalling). Recent comprehensive gene expression analyses of Arabidopsis (Arabidopsis thaliana), rice (Oryza sativa) and soybean (Glycine max) found that the ABRE is the most conserved of the dehydration-inducible promoters in these species, suggesting that the transcriptional regulation of dehydration inducible genes is similar among these species, with an ABRE-dependent transcriptional pathway (Nakashima and Yamaguchi-Shinozaki 2013). The expression level of drought- and ABA-responsive genes were increased under drought conditions in the transgenic plants (Cui et al., 2018). Consistent with previous findings, *ABA-insensitive protein 5*, and *ABA-responsive protein ABR18* were up-regulated with increase in drought time, indicating that it may be important for drought adaptation by affecting as yet unidentified mechanisms.

TFs of the family MYB, MYC, WRKY, bZIP, DREB (AP2/ERF), and NAC are widely known to respond to drought stress in plants. 416 TFs have been identified in our research including 37 AP2/ERF-ERF, 32 C2H2, and 22 bHLH, 22 bZIP, followed by C3H (19), MYB (18), WRKY (18), GRAS (16), and NAC (15). NAC TFs are involved in drought tolerance, and were explored in various crops like (Nakashima et al., 2012), rice (Shao et al., 2015), and soybean (Le et al., 2011). In our dataset, the expression of the 15 NAC transcription factor gene shows significant changes in the three comparisons. NAC transcription factor *PwNAC11* Activates *ERD1* by interaction with ABF3 and DREB2A to enhance drought tolerance in transgenic *Arabidopsis*. Genetic evidence demonstrated that *PwNAC11* physically interacted with an ABA-induced protein-ABRE Binding Factor3 (ABF3) and promoted the activation of promoter, which implied an ABA-dependent signaling cascade controlled by *PwNAC11* (Yu et al., 2021).

WRKY TF family members stand out among plant transcriptional regulators related to drought stress, which have been found in the cowpea WRKY gene family (*VuWRKY*) in response to water deficit (Matos et al., 2022). The overexpression of *TaWRKY1-2D* in transgenic *Arabidopsis* enhanced drought tolerance, which was accompanied by more lateral roots, lower stomatal aperture, and water loss (Yu et al., 2022). *ZmWRKY79* play the positive role in the drought-tolerance mechanism through regulating ABA biosynthesis, which provides a WRKY candidate gene to improve drought tolerance for maize and other crop plants (Gulzar and Fu 2021). Genes annotated as WRKY (18) had significantly decreased levels in 48h and 72 h relative to 0 h (Figure 4). Other drought-responsive TFs, such as MYB, bZIP, bHLH, and C2H2, showed differential expression under drought stress, as reported in other plant species (Yang et al., 2017). Collectively, the differential expression of various TFs families such as the one named above, ABA-responsive gene and their interaction with each other in a complex network crucially contributes to drought stress tolerance.

## Conclusion

In summary, we analyzed the full length Alfalfa transcriptome under drought stress for the first time, and identified specific drought-responsive genes and TFs. Our findings construct a sight of the gene modules involved in ABA or/and auxin biosynthesis during drought within a specific tissue and at a specific point in time. The information lays the foundation of future mechanistic and functional studies on drought-responsiveness in Alfalfa plants.

## Data Availability

The original contributions presented in the study are publicly available. This data can be found here: https://www.ncbi.nlm.nih.gov/sra/PRJNA531296.

## Supplementary material

Supplementary Figure S1 The data information of full-length transcriptome. **(A)** FLNC readlength distribution of each size bins. **(B)** FLNC read lengthdistribution for 1-6K size bins. **(C)** ROI classification for 1-6K size selected library.
