# Supplementary material for: Full-length transcriptome of in Medicago sativa L. roots in response to drought stress
Source: Front Genet. 2023 Jan 4;13:1086356. doi: 10.3389/fgene.2022.1086356 (PMC9848396; doi:10.3389/fgene.2022.1086356)
Supplement: Supplementary file 3 [file Table1.DOCX]

| **Annotated databases** | **Isoform Number** |
| --- | --- |
| COG | 32,297 |
| GO | 55,044 |
| KEGG | 34,349 |
| KOG | 48,647 |
| Pfam | 62,168 |
| Swiss-Prot | 54,521 |
| eggNOG | 72,017 |
| nr | 76,356 |
| All | 77,221 |

**Supplemental Table 1 Statistical table of annotated transcripts**
