# Supplementary figures and images for: Full-length transcriptome of in Medicago sativa L. roots in response to drought stress
Source: Front Genet. 2023 Jan 4;13:1086356. doi: 10.3389/fgene.2022.1086356 (PMC9848396; doi:10.3389/fgene.2022.1086356)

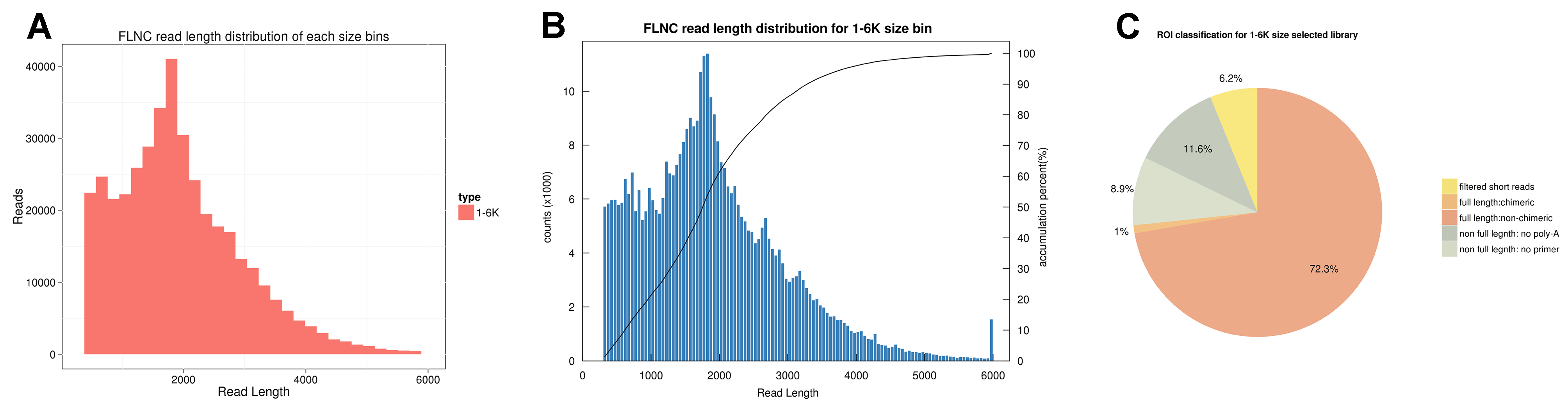

Supplement: Supplementary file 4 [file Image1.TIF]
